# Payor and patient costs for rapid brain MRI: analysis from a single pediatric institution

**DOI:** 10.1007/s00247-026-06648-5

**Published:** 2026-05-16

**Authors:** Shireen Hayatghaibi, Pradipta Debnath, Lisa Ulland, Brian Coley, Mishka Hoo Kim, Andrew Trout, Rama Ayyala

**Affiliations:** 1 Department of Radiology, Cincinnati Children’s Hospital Medical Center, 3333 Burnet Ave, Cincinnati, OH 45229 USA; 2 University of Cincinnati Medical Center, Cincinnati, USA; 3 Department of Medicine, Emory University, Atlanta, USA

**Keywords:** Brain, Magnetic resonance imaging, Deductibles and coinsurance, Policy

## Abstract

**Background:**

“Rapid” “Fast” or “Abbreviated” MRI examinations, which leverage fewer sequences to answer a targeted clinical question, are increasingly being explored as clinical tools.

**Objective:**

The purpose of this study was to evaluate and compare payor costs and patient out-of-pocket costs for brain MRI without contrast compared to a rapid brain MRI among commercially insured patients at a quaternary academic children’s hospital.

**Materials and methods:**

We performed a retrospective search to identify patients with private insurance who underwent an outpatient brain MRI without contrast (standard MRI) or rapid brain MRI at our quaternary academic children’s hospital. Examinations lacking complete payment information were excluded, and only the first exam per patient was included. All rapid MRIs were included; an equal number of standard MRIs were randomly sampled by month. For rapid MRI examinations, we calculated the frequency of cases in which the payor did not recognize the limited modifier, as indicated by the payor reason codes. For all examinations, we calculated the (1) payor cost (the amount reimbursed for the exam) and (2) the patient’s total out-of-pocket cost, calculated as the sum of the deductible, coinsurance, and co-payment. Descriptive statistics were used and means were compared between groups using Student’s *t* test.

**Results:**

Our sample included 147 standard MRIs and 166 rapid MRIs (coded with the 52 modifier). Most examinations included patient cost sharing: 77% (113/147) of standard MRIs and 69% of rapid MRIs (115/166). Payor reimbursement differed (*P*<0.001) by examination, with higher payor costs for standard MRI (mean, $2,760; SD, $1,187 vs. mean, $1,986; SD, $198) for rapid MRI. Among examinations with cost sharing, the mean total out-of-pocket costs were similar between examination types (standard, $1,206 vs rapid, $1,285; *P*=0.55). In 43% (71/166) of rapid MRI examinations, the payor did not recognize the limited modifier.

**Conclusion:**

Although rapid brain MRIs reduced payor reimbursement, patient out-of-pocket costs remained unchanged. Inconsistent recognition of the limited modifier underscores the need for updated CPT codes and reimbursement policies aligned with evolving imaging practices.

## Introduction

Magnetic resonance imaging (MRI) is a widely utilized modality in pediatrics, because of its high contrast resolution and its non-ionizing radiation. A major drawback of MRI is that it generally requires long examination durations and may necessitate the use of sedation or anesthesia to limit motion artifact. “Rapid” “Fast” or “Abbreviated” MRI examinations leverage fewer sequences to answer a targeted clinical question and are increasingly being implemented as clinical tools. Previous clinical studies have highlighted their use for various indications, such as appendicitis [1], breast imaging in adults [2], traumatic brain injury [3], and shunted hydrocephalus [4]. Rapid MRI examinations have enabled MRI utilization by facilitating faster patient throughput, negating the need for sedation, and reduced overall cost of the examination [5–7].

While clinical advances in rapid MRI continue, the financing and billing practices have lagged behind these advances. Despite growing utilization of rapid MRI, there is no distinct Current Procedural Terminology (CPT) code that distinguishes this type of MRI examination from a full diagnostic MRI examination. In the absence of a billing standard, institutions may vary in their billing practices. Some institutions submit claims and bill for full MRI exam, while others apply the modifier 52 which indicates a “reduced services” or a partial reduction in the MRI examination and a corresponding lower charge and reimbursement [8]. As a result, billing practices for rapid MRI lack consistency across health systems.

Coding variability is a function of the Centers for Medicare and Medicaid Services (CMS) payment policies, which directs reimbursement rates for CPT codes based on relative value units (RVUs). However, reimbursement standards for the 52 modifier are not explicitly defined. Because rapid MRI can be billed under the same CPT code as a complete MRI, it may be reimbursed at the same rate as a full diagnostic study. However, the clinical intent, acquisition time, resource use, and associated costs can differ substantially.

The lack of alignment between clinical practice and reimbursement policy contributes to wide variation in institutional billing approaches. Private insurers may establish their own reimbursement rules for limited examinations, adding further heterogeneity across geographical markets [9]. Despite the adoption of rapid MRI in pediatric settings, no real-world evidence describes how these examinations are reimbursed in practice, either by private payors or patient cost sharing. The purpose of this study was to evaluate and compare payor reimbursement and patient out-of-pocket costs for brain MRI without contrast compared to a rapid brain MRI among commercially insured patients at a quaternary academic children’s hospital.

## Methods

We hypothesized that the application of the limited modifier for rapid brain MRI would not consistently lead to lower patient out-of-pocket costs.

The institutional review board approved this retrospective study and waived the requirement for written informed consent. We performed a retrospective search to identify patients who underwent outpatient MRI of the brain without contrast (standard MRI) or rapid MRI of the brain between January 2024 and December 2024 at our quaternary academic children’s hospital. All financial information was retrieved from our institution’s electronic medical record. We excluded examinations covered by Medicaid, as these did not include patient cost sharing. During the study period, all eligible rapid MRI examinations were included. For standard brain MRI examinations, an equal number of patients per calendar month were randomly selected to match the rapid MRI cohort using the random number function in Microsoft Excel (Microsoft Corporation, Redmond, WA). Patients without complete payment information available in the electronic health record were excluded. For patients with multiple examinations during the year, we included only the first examination to avoid bias from lower or no cost sharing on subsequent examinations once deductibles were met.

For rapid MRI examinations, we calculated the frequency of cases in which the payor did not recognize the limited modifier, as indicated by the payor reason codes. For all examinations, we calculated the (1) payor reimbursement (the amount the insurer paid to the institution) and (2) the patient’s total out-of-pocket cost (the total costs the patient paid), calculated as the sum of the deductible (amount the patient pays before insurance pays), coinsurance (percentage of costs paid by patients after the deductible), and co-payment (a fixed cost the patient pays). Costs were inclusive of both technical and professional fees.

### Statistical analysis

Continuous variables were summarized descriptively using means and standard deviations and compared between groups using Student’s *t* test. Medians were compared using nonparametric tests, and categorical variables were compared using chi-square tests. A two-sided *P*-value <0.05 was considered statistically significant. The analysis was conducted using Stata EP version 17 (StataCorp LLC).

## Results

Our sample included 147 standard MRIs and 166 rapid MRIs which were billed with 52 modifiers (Table 1). Overall, seasonality (by quarter) and insurance payors did not differ significantly between groups.

**Table 1:** Patient and examination characteristics for standard and rapid MRI

	Standard (*n*=147)	Rapid (*n*=166)	*P****-***value
Age (years), mean (SD)	12.8 (4.4)	6.9 (6.2)	<0.001
Female, *n* (%)	94 (64.0)	63 (38.0)	<0.001
Quarter, *n* (%)			0.79
Jan-Mar	42 (28.6)	42 (25.3)	
Apr-Jun	34 (23.1)	36 (21.7)	
Jul-Sept	29 (19.7)	32 (19.3)	
Oct-Dec	42 (28.6)	56 (33.7)	
Insurance, *n* (%)			0.16
Anthem	83 (56.5)	104 (62.7)	
United	48 (32.7)	44 (26.5)	
Cigna	5 (3.4)	8 (2.8)	
Aetna	4 (2.7)	-	
Misc	7 (4.8)	10 (6.0)	
Cost sharing, *n* (%)	113 (76.9)	115 (69.3)	0.13

### Payor reimbursement

Patient out-of-pocket cost sharing occurred in 77% (113/147) of standard MRIs and 69% (115/166) of the rapid MRIs. Payor reimbursement differed significantly by examination type (*P* <0.001), with higher mean reimbursement for standard MRI compared with rapid MRI (mean $2,760, SD $1,187 vs. mean $1,986, SD $198). Reimbursement also varied across payors (Table 2). Among examinations that involved patient cost sharing, the total payor payment remained significantly higher for standard MRI than for rapid MRI (mean $2,628 vs. $1,642; *P* <0.001).

**Table 2:** Payor reimbursement and patient out-of-pocket costs for standard vs rapid MRI

	Standard	Rapid	*P****-***value
Mean, total payor reimbursement	$2,760	$1,986	<0.001
Median, total payor reimbursement	$3,138	$1,972	<0.001
Mean, OOP cost (all observations)	$988 (*n*=147)	$835 (*n*=166)	0.18
Mean, OOP cost (OOP cost >0)	$1,285 (*n*=113)	$1,206 (*n*=115)	0.55
Median, OOP cost (OOP cost >0)	$831 (*n*=113)	$800 (*n*=115)	0.57

*OOP*, out-of-pocket

### Patient out-of-pocket costs

**Fig. 1:**
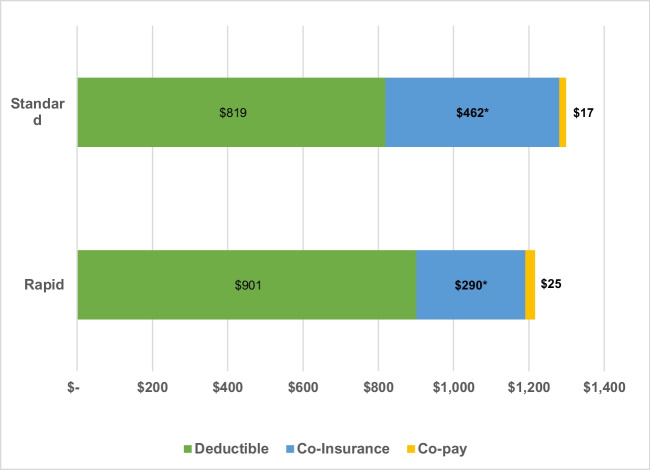
Patient cost-sharing component for standard and rapid MRI. *Deductible*, amount the patient pays before insurance pays; *coinsurance*, percentage of costs paid by patients after the deductible; *co-pay*, a fixed cost the patient pays. Coinsurance differed significantly between groups (*P*=0.001); no significant differences were observed for deductible or co-payment amounts

Despite differences in payor reimbursement, patient out-of-pocket costs did not differ significantly between standard and rapid MRI examinations. Among examinations with cost sharing, the mean total out-of-pocket costs were similar between examination types (standard, $1,206 vs rapid, $1,285; *P*=0.55). The median out-of-pocket costs were also comparable (standard, $831 vs rapid, $800; *P*=0.57). Across all examinations, deductibles accounted for the largest share of patient out-of-pocket costs, followed by coinsurance, while fixed co-payments contributed minimally (Fig. 1). Among out-of-pocket cost components, deductible and co-payment costs were similar; however, coinsurance was significantly higher for standard MRI than for rapid MRI (mean $462 vs $290; *P*=0.001).

Among rapid MRI examinations, the payor did not recognize the limited modifier in 43% (71/166) of these claims. Modifier recognition varied across commercial payors, with some consistently recognizing the limited modifier and others rarely recognizing it. Mean out-of-pocket costs were lower when the limited modifier was recognized ($1,042) compared with when it was not recognized ($1,417; *P*=0.03). When the modifier was not recognized, reimbursement was comparable to standard diagnostic MRI.

## Discussion

Our institutional findings demonstrate that while rapid brain MRI examinations billed with a limited modifier are associated with significantly lower payor reimbursement compared to standard brain MRI, these lower costs do not translate to significantly lower patient out-of-pocket costs, unless the payor recognizes the limited modifier. These findings were consistent with our hypothesis that use of the limited modifier for rapid MRI would not reliably result in lower patient out-of-pocket costs. Instead, the patient out-of-pocket costs are driven by insurance benefit design, which is influenced by timing of the year. For instance, we found that the cost sharing is mainly due to deductibles. While we did not have access to the patient’s specific benefit plan design, we suspect that high-deductible plans may be a factor in these costs, thus even when rapid MRI is reimbursed at a lower rate, patients continue to face similar cost sharing obligations.

Additionally, we observed that the limited modifier was not recognized by payors in 43% of rapid brain MRI examinations, resulting in reimbursement rates and patient cost sharing that were similar to a standard MRI. This inconsistency undermines the intended purpose of the limited modifier, ultimately resulting in patients incurring similar out-of-pocket costs for examination coded as reduced imaging services. This suggests a need for coding standards, such as the creation of a distinct CPT code that reflects changing imaging practices. As rapid MRI continues to expand in clinical use, policy and reimbursement frameworks must also adapt. Future studies should evaluate the patient out-of-pocket costs for brain MRI using national claims data to assess the generalizability of these findings.

The purpose of the limited modifier is to indicate that a reduced service was provided and thus reduced reimbursement is warranted. This reduction in reimbursement can be justified by the reduced facility costs of performing a rapid MRI [10]. However, a recent report by Huang et al. demonstrated declines in CMS reimbursement for MRI over the past two decades [11]. The authors argued that the general reduction in CMS reimbursement precludes further reduction in reimbursement for rapid MRI examinations because fixed costs limit facility cost savings from shorter exam times.

While their analysis focuses on public insurance reimbursement trends, our findings in commercial payors highlight that lower reimbursement for rapid brain MRI does not always translate into significantly lower patient out-of-pocket costs. This likely reflects the complexity of insurance benefit design, which includes high-deductible health plans where patients are responsible for higher cost sharing amounts. Additionally, inconsistent payor recognition of the limited modifier resulted in non-discounted reimbursement and patient cost sharing for a substantial proportion of rapid MRI examinations. Together, these findings highlight a disconnect between reimbursements and patient cost sharing, suggesting that existing coding mechanisms may inadequately be recognized across payors. These dynamics may also help explain the rise of insurer-driven steerage programs, where insurers direct patients to lower-cost providers. However, when out-of-pocket costs do not reflect underlying price differences, patients have limited ability to respond to price signals, particularly when price transparency is limited. Although lower reimbursement for rapid MRI did not reduce patient out-of-pocket costs in this study, it may yield indirect benefits for beneficiaries in self-funded plans where reduced spending can translate to lower premiums over time, though these effects are indirect and not guaranteed.

While there is debate about the appropriateness of reducing charges to payors for rapid MRI examinations, we suspect there may be less debate about reducing costs for patients. Unfortunately, inconsistent recognition of the limited modifier suggests that current coding approaches may not reliably translate reduced imaging services into predictable reductions of patient cost sharing. Future work should examine whether alternative coding strategies, including a dedicated CPT code, could more consistently translate to cost savings to patients.

Limitations of this study include the fact that this analysis was conducted at a single quaternary academic children’s hospital and results may not be generalizable. Our findings on payor recognition variability of the limited modifier likely reflect broader challenges in billing across commercial insurers. We did not have access to the patient’s detailed insurance benefit design information, such as maximum deductible thresholds, coinsurance rates, or timing within the benefit year. Finally, this study was restricted to brain MRI examination; thus, our findings may not be generalizable to other types of rapid MRI examinations.

Rapid MRI protocols have demonstrated benefits in reducing sedation, avoiding ionizing radiation, and improving patient throughput, particularly in pediatric populations. The lack of effective reduction of out-of-pocket costs through use of a limited modifier does not diminish this clinical value. However, under current reimbursement structures, the financial efficiencies gained are primarily recognized at the system and payor level, rather than to patients.

## Data Availability

The data underlying this study are not publicly available because of IRB and patient privacy restrictions.
